# A Comparative Study of Two Fractional-Order Equivalent Electrical Circuits for Modeling the Electrical Impedance of Dental Tissues

**DOI:** 10.3390/e22101117

**Published:** 2020-10-03

**Authors:** Norbert Herencsar, Todd J. Freeborn, Aslihan Kartci, Oguzhan Cicekoglu

**Affiliations:** 1 Department of Telecommunications, Brno University of Technology, Technicka 3082/12, 616 00 Brno, Czech Republic; kartci@feec.vutbr.cz; 2 Department of Electrical and Computer Engineering, The University of Alabama, Tuscaloosa, AL 35487, USA; tjfreeborn1@eng.ua.edu; 3 Department of Electrical and Electronics Engineering, Bogazici University, Bebek, Istanbul 34342, Turkey; cicekogl@boun.edu.tr

**Keywords:** bioimpedance, biomedical tissue, Cole–Cole model, constant phase element, CPE, electrical impedance spectroscopy, EIS, fractional calculus, human tooth dentin model, Valsa method

## Abstract

*Background:* Electrical impedance spectroscopy (EIS) is a fast, non-invasive, and safe approach for electrical impedance measurement of biomedical tissues. Applied to dental research, EIS has been used to detect tooth cracks and caries with higher accuracy than visual or radiographic methods. Recent studies have reported age-related differences in human dental tissue impedance and utilized fractional-order equivalent circuit model parameters to represent these measurements. *Objective:* We aimed to highlight that fractional-order equivalent circuit models with different topologies (but same number of components) can equally well model the electrical impedance of dental tissues. Additionally, this work presents an equivalent circuit network that can be realized using Electronic Industries Alliance (EIA) standard compliant RC component values to emulate the electrical impedance characteristics of dental tissues. *Results:* To validate the results, the goodness of fits of electrical impedance models were evaluated visually and statistically in terms of relative error, mean absolute error (MAE), root mean squared error (RMSE), coefficient of determination ($R^{2}$), Nash–Sutcliffe’s efficiency (NSE), Willmott’s index of agreement (WIA), or Legates’s coefficient of efficiency (LCE). The fit accuracy of proposed recurrent electrical impedance models for data representative of different age groups teeth dentin supports that both models can represent the same impedance data near perfectly. *Significance:* With the continued exploration of fractional-order equivalent circuit models to represent biological tissue data, it is important to investigate which models and model parameters are most closely associated with clinically relevant markers and physiological structures of the tissues/materials being measured and not just “fit” with experimental data. This exploration highlights that two different fractional-order models can fit experimental dental tissue data equally well, which should be considered during studies aimed at investigating different topologies to represent biological tissue impedance and their interpretation.

## 1. Introduction

The interest in the application of fractional calculus, the branch of mathematics concerning integration and differentiation to non-integer orders, to modeling complex biomedical phenomena has increased significantly in recent decades. This interest stems from the fitting accuracy achieved between experimental data and fractional-order models [1]. In many cases the fractional-order models utilize fewer parameters than the integer-order models traditionally used; offering an opportunity for reduced order modeling without decreases in fitting accuracy. The integro-differential operator Dtαa with a,t∈R and α∈R (where α is the fractional-order of the operation) is defined as follows:

(1) Dtαa=dαdtα:α>0,1:α=0,∫at(dτ)−α:α<0.

To evaluate a fractional-order derivative or integral of a function, three different definitions are used [2,3]:

- Riemann–Liouville and Caputo for continuous-time domain,
- Grünwald–Letnikov in the discrete domain.

The Caputo definition is widely adopted because the initial conditions for this definition are described in the same form as integer-order differential equations [4]. For reference, Caputo’s derivative is described as:

(2) DtαaCf(t)=1Γ(n−α)∫atf(n)(τ)(t−τ)α−n+1dτ,

where n∈N:n−1<α≤n and Γ(·) is the Euler’s Gamma function [3]. Applying the Laplace transform to Caputo’s derivative with a=0 yields:

(3) LDtα0Cf(t)=sαF(s)−∑k=0n−1sα−k−1f(k)(0).

Considering the case where the initial conditions in (3) are zero, the form in (3) can be rewritten as follows:

(4) LDtα0Cf(t)=sα·F(s).

This concept can be applied to the impedance properties of electrical circuit elements to define a general fractional-order element (FOE) with impedance proportional to the Laplacian operator ($s^{\alpha }$), where ω is the angular frequency with s=jω. Note that, in this case, the impedance phase is a function of the fractional-order, given in radians as ϕ = απ/2 or in degrees as ϕ = 90$\alpha^{\circ }$. From the general FOE, it is possible to define elements with characteristics between the traditional components of resistors, capacitors, and inductors. For example, a fractional-order capacitor (FOC) and fractional-order inductor (FOI) can be defined. The impedance of an FOC ($Z_{\mathrm{FOC}}=1/s^{\alpha }C_{\alpha }$) is limited to fractional-orders 0 < α < 1 and a pseudo-capacitance $C_{\alpha }$ with units Farad·$\sec^{\alpha -1}$ (F·$s^{\alpha -1}$). Similarly, an FOI will have an impedance ($Z_{\mathrm{FOI}}=s^{\alpha }L_{\alpha }$) with fractional-order limited to 0 < α < 1 and a pseudo-inductance of $L_{\alpha }$ with units expressed as Henry·$\sec^{\alpha -1}$ (H·$s^{\alpha -1}$) [5,6,7]. From the impedance definitions of the FOC and FOI, the frequency dependent impedance magnitude varies by −20α dB/decade and +20α dB/decade of frequency, while the phase is constant with frequency (though dependent on the fractional-order) and equal to ±απ/2. It is for this constant phase behavior that these devices are often referred to as constant phase elements (CPEs).

One of the application areas of fractional calculus is modeling the electrical impedance of biomedical tissues, also referred to as the tissue bioimpedance [8]. Bioimpedance is a complex quantity, which varies with the tissue’s composition (tissue type, cellular membrane integrity, intercellular and extracellular fluids) and the frequency of the electrical signal applied for impedance measurement. The frequency dependent impedance is represented generally in the forms:

(5)Z(jω)=V(jω)I(jω),(6)=|Z(jω)|·ejθ,(7)=Re(Z)+j·Im(Z),

where the real (Re(*Z*) or resistance *R*(Ω)) and imaginary (Im(*Z*) or reactance *X*(Ω)) parts of the complex impedance are calculated as R=|Z|·cosθ and X=|Z|·sinθ, respectively. The study of materials using their electrical impedance, referred to as electrical impedance spectroscopy (EIS) [9], is a powerful technique that measures the electrical impedance devices or materials (including biological tissues). The electrical impedance of a sample is measured by applying an electrical stimulus (such as a step function, noise signal, sinusoidal signal, chirp signal, or binary multi-frequency signals of voltage or current) and measuring the excited response (current or voltage). The impedance is then calculated using the current/voltage values as shown in (5) to (7) [10]. To date, EIS has been used in a broad range of applications including the characterization of neointimal tissue for stent applications, biological analysis and food characterization, detection of cells in suspensions, milk characterization, biceps tissue modeling, evaluation of wet aged beef, and determination of leaf nitrogen concentrations [5,11,12,13,14,15,16,17,18] (and references cited therein). While this list is not exhaustive, it does highlight the wide range of applications that EIS is being explored to support across medicine and food processing.

**Figure 1:**
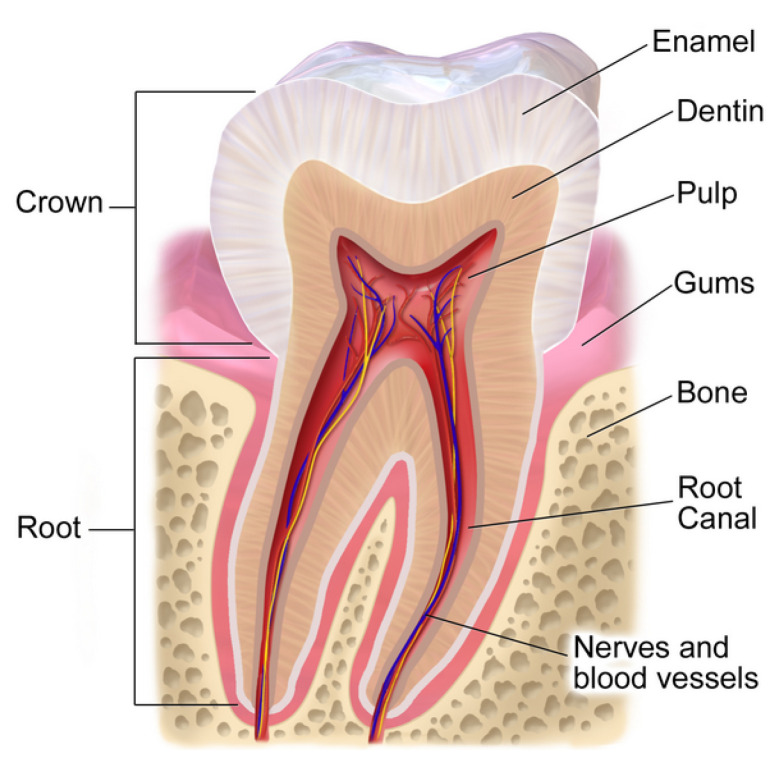
Tooth anatomy [19].

Another medical focused application of EIS is found in dentistry (the branch of medicine focused on studying, diagnosing, treating, and preventing diseases or disorders of the oral cavity) and the non-invasive assessment of dental tissues. The tooth, with dental anatomy depicted in Figure 1 [19], is the hardest substance in the human body [20] with roles for both eating and speech production. From the anatomy, the white outer layer of the crown of the tooth (known as the enamel) is mostly made of calcium phosphate, a rock-hard mineral [20]. Encased by the enamel is the dentin structure, which is a complex hydrated composite and constitutes the bulk of a human tooth [21]. In cases in which the enamel is damaged or decayed, pain, infection, and even tooth loss can occur [20]. The early detection of dental caries (or tooth decay) is a critical activity towards supporting oral health, which provides the motivation behind non-invasive methods for their detection. In several in vitro and in vivo studies, it has been shown that early detection of dental caries poses a major challenge. EIS has recently being investigated for this application [22,23,24,25,26,27]. Using EIS as a diagnostic tool requires determining a marker in the collected data that is strongly associated with the target pathology. Many studies are exploring equivalent electrical circuit parameters that represent EIS data for their association with dental caries. This approach requires that the experimental data are fit to a predetermined electrical circuit model which often incorporate a CPE (a fractional-order circuit element). Electrical circuit models employing CPEs that have been used in studies of the impedance characteristics of dental tissues are given in Table 1. In particular, significant attention has been given to using EIS to determine necessary root canal length measurements as well as the characterization of enamel and dentin structures. A root canal is a dental procedure to remove the infected tooth pulp when it gets infected due to a cavity or trauma. Hence, the success of endodontic treatment depends on the accurate measurement of a tooth root canal length. Various approaches have been employed to locate the canal apex of a diseased tooth [28]. Nowadays, electronic apex locators are used that are based on electrical impedance measurements. In [29], eight equivalent circuit models were compared for measuring root canal length. The most accurate model employed a single CPE in parallel with a series connection of CPE and a resistor. Other studies have: utilized models with a single CPE, two capacitors, and two resistors to represent measurements of tooth enamel [30]; utilized a four element circuit with three-series resistors in parallel with a CPE to represent measurements of dentin [31]; and utilized a model with three resistors and two CPEs during investigations of age related differences in dentin [32]. These highlight the wide range of electrical equivalent models that have been used and for a range of dental applications. The selection of the equivalent electrical circuit to use during the fitting of impedance elements is an important consideration. Often the aim is to utilize a model that has the least number of parameters, but is grounded in the underlying structure of the tissue so that model parameters have a physical interpretation. Determine the most appropriate circuit models for different applications is an active area of EIS, and motivates the work in this study.

In this paper, two fractional-order circuit models are explored to represent previously collected electrical impedance data of teeth dentin (first reported in [32], but numerically reconstructed for use in this work). The purpose of this work was to illustrate that different topologies with the same number of components can equally well represent electrical impedance data, highlighting a challenge of equivalent circuit modeling that should be considered during study design, data analysis, and study comparisons. Second, this study reports an empirical teeth dentin model that can be constructed using Electronic Industries Alliance (EIA) compliant components to emulate the fractional-order model impedance. The goodness of fit of the proposed empirical electrical models of CPEs and emulated fractional-order equivalent electrical circuits are compared using assessments of mean absolute error, room mean squared error, and coefficients of determination. Temperature variation, Monte Carlo statistical analysis, and thermal noise voltage variations of the proposed recurrent circuit models were simulated in the frequency domain via SPICE software under practical design aspects. The use of the constructed models is expected to support further studies where measurements or simulations representative of dental tissues may be needed to characterize measurement equipment, but fractional-order components are not available for their realization or simulation.

## 2. Materials and Methods

### 2.1. Human Teeth Dentin Samples Preparation and Data Collection

The experimental data that was reconstructed for further analysis in our study was first reported by Eldarrat et al. [32]. We refer readers to this work for the the complete details of the experimental EIS configuration for data collection (sample preparation, sample holder, physiological saline solution, and impedance of a control electrical). As a summary of the data collection process, Eldarrat et al. reported that freshly extracted un-erupted human third molars were used to avoid the effect of attrition due to age [32]. Immediately after extraction, soft tissue debris and bone fragments were removed and the teeth stored in hermetically sealed vials containing physiological saline with a few Thymol crystals. Two age groups were selected in the investigation; 20 (±1) and 50 (±1) years old and five dentin samples were collected from each age group. Bioimpedance measurements were carried out at 20 °C using a Solartron Analytical SI 1260 Impedance Gain-Phase Analyzer over the frequency range 10 mHz to 10 MHz. The applied amplitude of the ac potential was 100 mV rms under open circuit conditions. Coaxial leads were used to connect the sample to the analyzer and these leads were kept as short as possible to minimize stray capacitance. Note that the dental tissues reported by Eldarrat et al. [32] were not utilized directly in this work, and instead the reported impedance of the dental tissues in [32] was utilized for this secondary analysis.

### 2.2. The Double Dispersion Cole Bioimpedance Model and Data Reconstruction

**Figure 2:**
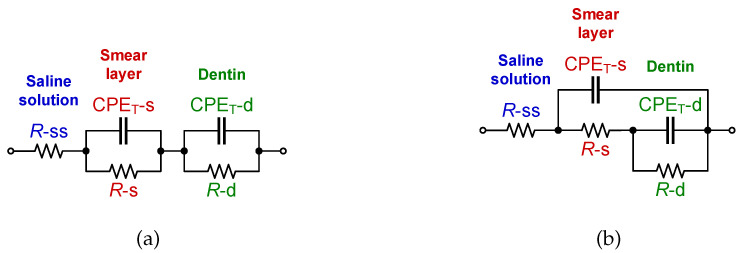
(**a**) Double dispersion Cole impedance model, (**b**) recurrent electrical impedance model for n=2 bifurcations.

In [32], Eldarrat et al. proposed that the fractional-order circuit model depicted in Figure 2a provided the best fit in terms of accuracy for their experimental data. This model has been previously referred to as the double-dispersion Cole impedance model and will be referred to as the C-C model in this work. Using this model, Eldarrat et al. proposed that the smear layer over the dentin surface and dentin itself have their own resistance (respectively *R*-s, *R*-d) and pseudo-capacitance (CPE_T_-s, CPE_T_-d), while the saline solution is purely resistive (*R*-ss). The impedance in terms of the parallel/series combinations of elements of the C-C model is given by:

(8) $$Z_{C-C}=Z-\mathrm{ss}+\left(Z_{\mathrm{CPE}}-s||Z-s\right)+\left(Z_{\mathrm{CPE}}-d||Z-d\right),$$

where Z-k=R-k for k∈(ss,s,d), $Z-l\left(s\right)=1/s^{{\mathrm{CPE}}_{P}-l}\cdot {\mathrm{CPE}}_{T}-l$ for l∈(s,d) while 0 < ${\mathrm{CPE}}_{P}-l$ < 1, and taking into account equal orders of CPEs used (α = CPE_P_-s = CPE_P_-d = CPE_P_). Therefore, the impedance of the C-C model using fractional-circuit theory [33] becomes:

(9) $$Z{\left(s\right)}_{C-C}=R-\mathrm{ss}+\frac{1}{s^{{\mathrm{CPE}}_{P}}\cdot {\mathrm{CPE}}_{T}-s+\frac{1}{R-s}}+\frac{1}{s^{{\mathrm{CPE}}_{P}}\cdot {\mathrm{CPE}}_{T}-d+\frac{1}{R-d}},$$

where s=jω. The complex impedance of (9) can be calculated using the replacement $s^{{\mathrm{CPE}}_{P}}=\omega^{{\mathrm{CPE}}_{P}}\left[\cos\left(\frac{{\mathrm{CPE}}_{P}\cdot \pi }{2}\right)+j\cdot \sin\left(\frac{{\mathrm{CPE}}_{P}\cdot \pi }{2}\right)\right]$. From (9), the impedance at ω=0 reduces to $Z_{C-C}=R-\mathrm{ss}+R-s+R-d$ and $Z_{C-C}=R-\mathrm{ss}$ at ω=∞; entirely resistive at the theoretical low and high frequencies.

**Figure 3:**
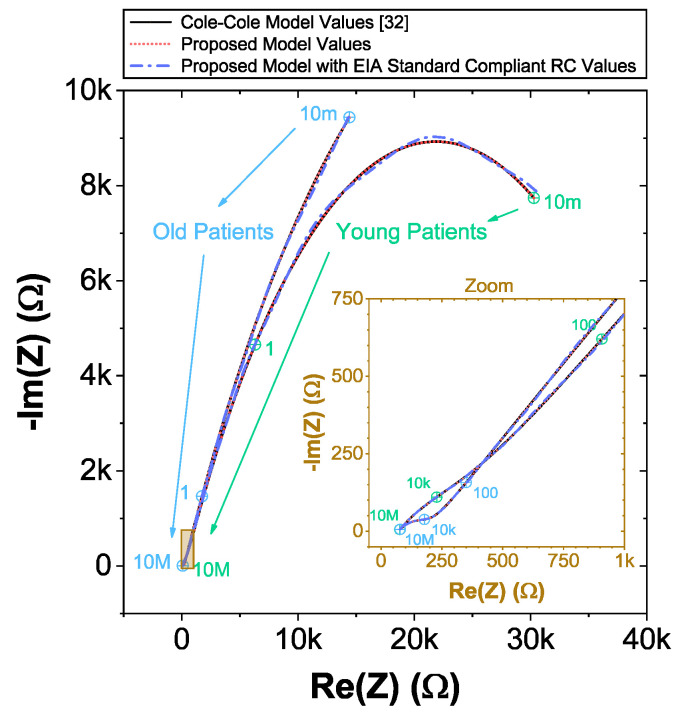
Nyquist plots of simulated [32] and proposed recurrent circuit models of young and old patients dentin with zoom at mid and high frequency region as an inset. Selected frequencies are highlighted.

The complex impedance data of the C-C model that is used in the following sections was numerically reconstructed from the two age groups using the mean dental tissue values reported in [32] (given in Table 2 for reference). These reconstructed datasets were generated using (9) in the frequency range 10 mHz ≤ω/2π≤ 10 MHz with 1400 logarithmic space points. For visual reference, these reconstructed datasets are given in Figure 3 as solid lines. Note the significant difference between these datasets at low and high frequencies, highlighting the difference in impedance of different age groups reported in [32].

### 2.3. Proposed Recurrent Electrical Impedance Model with Optimal Values

While the C-C model was previously utilized to represent the dental-tissue impedance data, other fractional-order model topologies should be able to also represent the impedance datasets. Using different topologies to represent impedances from similar tissues prevents direct comparison of circuit parameters between datasets and introduces the opportunity to utilize topologies that may not be directly linked to tissue structure or physiological features. To highlight this, this work fits both the C-C model and a recurrent electrical impedance model for n=2 bifurcations to reconstructed dental tissue impedance data. The recurrent model is shown in Figure 2b [4]. Similar to the C-C model in Figure 2a, this model consists of three resistors and two CPEs; therefore it is expected to be able to model the same dental tissue quantities, i.e., resistance and pseudo-capacitance of smear layer and dentin as well as the saline solution resistance. The general impedance form of the recurrent electrical impedance model for n=2 bifurcations is:

(10) $$Z_{\mathrm{Rec}-2}=Z-\mathrm{ss}+\left[Z_{\mathrm{CPE}}-s||\left\{Z-s+\left(Z_{\mathrm{CPE}}-d||Z-d\right)\right\}\right],$$

where Z-k=R-k for k∈(ss,s,d), $Z-l\left(s\right)=1/s^{{\mathrm{CPE}}_{P}-l}\cdot {\mathrm{CPE}}_{T}-l$ for l∈(s,d), while 0 < ${\mathrm{CPE}}_{P}-l$ < 1, and equal order CPEs are used (α = CPE_P_-s = CPE_P_-d = CPE_P_). The impedance of this model can also be described by:

(11) $$\begin{array}{cc} Z{\left(s\right)}_{\mathrm{Rec}-2}=R-\mathrm{ss}+\frac{1}{s^{{\mathrm{CPE}}_{P}}\cdot {\mathrm{CPE}}_{T}-s+\frac{1}{R-s+\frac{1}{s^{{\mathrm{CPE}}_{P}}\cdot {\mathrm{CPE}}_{T}-d+\frac{1}{R-d}}}} & , \end{array}$$

where s=jω. The complex impedance of (11) can be expressed using the replacement $s^{{\mathrm{CPE}}_{P}}=\omega^{{\mathrm{CPE}}_{P}}\left[\cos\left(\frac{{\mathrm{CPE}}_{P}\cdot \pi }{2}\right)+j\cdot \sin\left(\frac{{\mathrm{CPE}}_{P}\cdot \pi }{2}\right)\right]$. Note that the impedance at ω=0 and ω=∞ match the C-C circuit model, i.e., $Z_{\mathrm{Rec}-2}{|}_{\omega \to 0}=R-\mathrm{ss}+R-s+R-d$ and $Z_{\mathrm{Rec}-2}{|}_{\omega \to \infty }=R-\mathrm{ss}$, respectively.

To determine the component values of the recurrent electrical impedance model for n=2 bifurcations that best fits the reconstructed data, a parametric weighted algorithm of Powell complex nonlinear least-squares (CNLS) fitting was used [9,34]. In this fitting method, the sum of the squares of real and imaginary residuals is minimized.

## 3. Results and Discussion

### 3.1. Comparison of Proposed Bioimpedance Models

The CNLS computed component values for the recurrent model are listed in Table 2. For comparison to the reference C-C reconstructions, simulations using these parameters in (11) are given in Figure 3 (as a dotted line). Visually, these simulations show very good agreement with each other with selected frequencies highlighted. Comparing the individual component values, notice the differences of the smear layer and dentin resistances and pseudo-capacitances. For example, CPE_T_-s for the young-patients have values of 23.8 $\mu F\cdot \sec^{-0.5}$ and 15.64 $\mu F\cdot \sec^{-0.5}$ for the C-C model and recurrent model, respectively. Highlight the differences in model parameter values that utilizing different topologies can yield even with good overall agreement for the impedance for both models. This also serves to reinforce that comparing model parameters of different equivalent circuits fit to impedance datasets should be carefully considered during evaluation of data from multiple studies or meta-analyses.

To quantify the fit of the recurrent model to the reconstructed C-C model electrical impedance, various statistical metrics [35] were evaluated including: max/min/median/standard deviation of relative differences, the mean absolute error (MAE—the sum of absolute values of the errors divided by the number of observed data points), the root mean squared error (RMSE—the square root of the average of squared errors), and the coefficient of determination ($R^{2}$—indicates the fraction of the fitting values that are closest to the line of reference data). These were calculated for both the real and imaginary components of the impedance, with metrics for each given in Table 3 and Table 4. Note that while the ideal value of statistical indicators MAE and RMSE is 0 (the lower value is the better value), a coefficient of determination close to 1 (or 100%) indicates a perfect fit. In addition, the overall performance of proposed models were evaluated by calculating the Nash–Sutcliffe’s efficiency (NSE—normalized statistic that determines the relative magnitude of the residual variance compared to the reference data variance), the Willmott’s index of agreement (WIA or index of agreement *d*—represents the ratio of the mean square error and the potential error), and Legates’s coefficient of efficiency (LCE—less sensitive to high extreme values, because uses absolute value of the difference instead of using the squared differences). LCE and NSE vary between 1 for perfect agreement and −∞ for complete disagreement, whereas WIA lies between 0 (no correlation) and 1 (or 100%; perfect fit). Using all of these metrics, a perfect agreement between the reconstructed C-C data and recurrent model are noted. This is not unexpected since the data utilized in the CNLS are ideal, that is without any noise contributions or deviations that would be expected from a measured dataset, but it still serves to highlight that both fractional-order models are able to represent the same ideal datasets. Building on this, future studies should investigate the differences in fittings of both models applied to experimental bioimpedance datasets of dental tissues and possible differences in fit towards advancing which model warrants further integration into dental tissue studies. Explore if there are features of experimental datasets that one model may capture more accurately than the other. Further, a deeper investigation into the physiological structure of dental tissues and comparison to the proposed models in this work should be investigated to determine which model most closely aligns with the tissue structure and will have the most meaningful clinical interpretations. For example, which circuit parameters are directly associated with changes in dentin and smear structure and which are strongly associated with tissue geometry? This is especially critical to translate these results from a theoretical exploration of circuit theory to an application with clinical value.

### 3.2. Empirical Electrical Model of CPEs via Valsa Method

**Figure 4:**
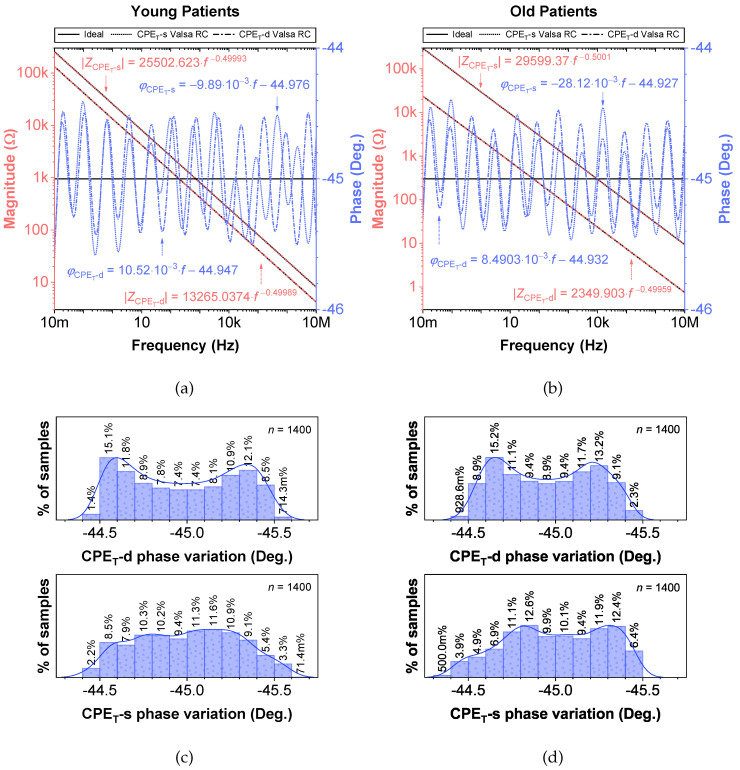
Proposed CPEs of order 0.5 via the Valsa method with ideal and simulated phase and magnitude responses vs. frequency: (**a**) young, (**b**) old patients teeth dentin, and (**c**,**d**) corresponding phase angle variations.

**Figure 5:**
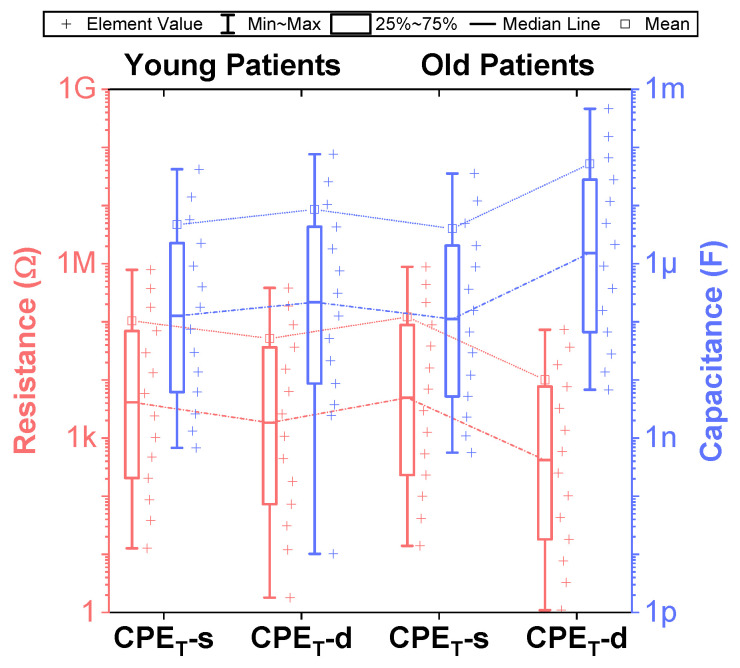
Distribution of resistance and capacitance values in the Valsa RC network for realizing constant phase elements of order 0.5 in the frequency range of 10 mHz–10 MHz.

While fractional-order circuit models can be evaluated for their “fit” and representation of electrical impedance data, there are no commercially available fractional-order elements available for their physical realization. Until fractional-order components are readily available, realizing these circuits for SPICE simulations and measurement requires the approximation of the fractional-order elements in these models. To support researchers interested in using an emulated model of dental tissues, the CPEs (adjusted to their nearest EIA standard compliant values) in Table 2 were realized using the Valsa method to realize an RC network with 13 branches [7,36] that approximates the CPE impedance over a fixed frequency band. This electric network is shown in Table 5 for reference. The resistance and capacitance values for this network were computed using the approach detailed in [37] powered by a genetic algorithm. This approach, based on a defined fitness function provides a phase optimization in the desired bandwidth (10 mHz–10 MHz) without any complex mathematical analysis. Table 5 lists the standard EIA compliant RC values used to realize the proposed CPEs with orders of 0.5. The variations of the approximated CPE phase and magnitude for the young and old patients’ teeth dentin compared to the ideal cases and corresponding phase angle changes in form of histograms are given in Figure 4. Further, the fitting equations using a power regression for the magnitudes and linear regression for the phases are given in Figure 4a,b. The coefficient of variation (CoV), which is the relative standard deviation (SD) expressed in percentage, was calculated as $\mathrm{CoV}=100\cdot SD/\tilde{x}$. Here, $\tilde{x}$ is the mean values of phase angle (order) or pseudo-capacitance values of CPEs. From these values, given in Table 5, the maximum absolute CoV indicators for phase angle (i.e., order) and pseudo-capacitance values of CPEs of young and old patients’ teeth dentin are 0.700%/0.631% and 3.503%/2.946%, respectively. Hence, the phase angle (order) and pseudo-capacitance mean values of CPEs are close to their theoretical values with low SD. In addition, both absolute relative error of magnitude and absolute phase angle error were calculated for the CPEs approximated using the Valsa approach. The maximum relative magnitude errors of CPEs of young and old patients teeth dentin are equally ±1.188% with maximum phase angle error $\pm 0.623^{\circ }$ and $\pm 0.606^{\circ }$, respectively, over frequency range of 10 mHz–10 MHz. These support that the approximated CPEs are a very good representation of the ideal CPEs. To further visualize the range of components required to realize these approximated CPEs, the distribution of resistance and capacitance values for both age groups are also depicted in Figure 5.

**Figure 6:**
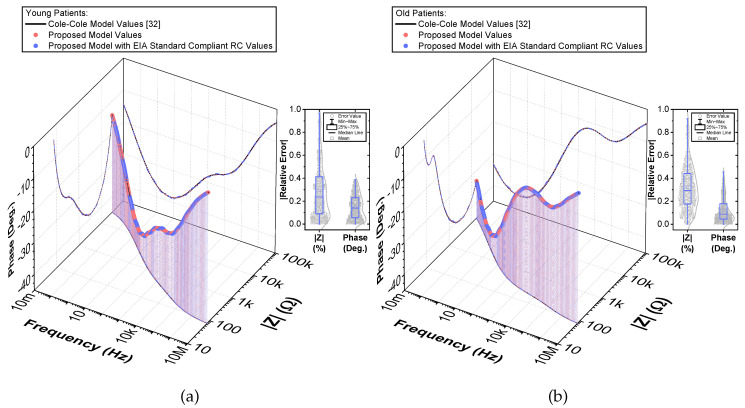
(**a**) Young and (**b**) old patients’ teeth dentin: 3D Bode plots of simulated [32] and proposed recurrent circuit models and distribution of absolute relative magnitude and phase angle errors of the model with EIA standard compliant RC values.

**Figure 7:**
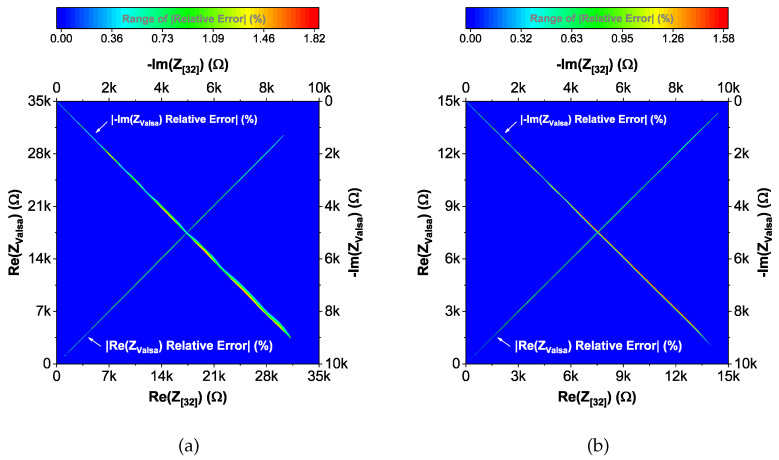
(**a**) Young and (**b**) old patients’ teeth dentin: Distribution of absolute relative errors reported in Table 3 of the proposed recurrent model with EIA standard compliant RC values.

**Figure 8:**
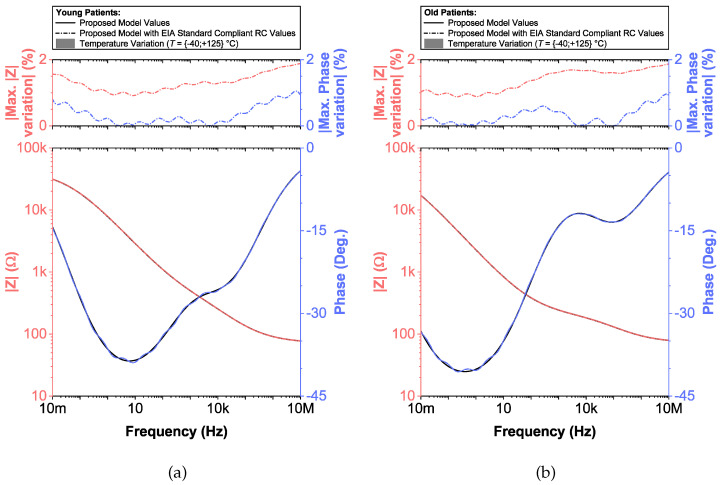
(**a**) Young and (**b**) old patients’ teeth dentin: Temperature variation of proposed recurrent circuit models with EIA standard compliant RC values (bottom plots) and distribution of maximum absolute relative magnitude and phase angle variation (top plots) vs. frequency.

**Figure 9:**
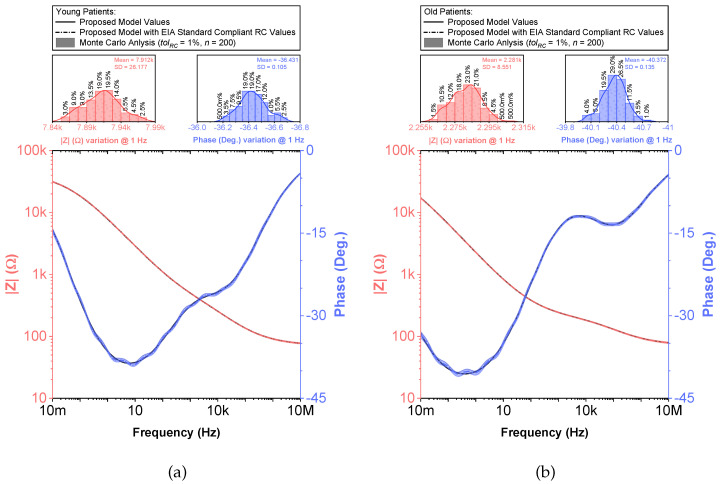
(**a**) Young and (**b**) old patients’ teeth dentin: Monte Carlo statistical analysis of proposed recurrent circuit models with EIA standard compliant RC values (bottom plots) and magnitude and phase angle variation at 1 Hz (histograms) vs. frequency.

**Figure 10:**
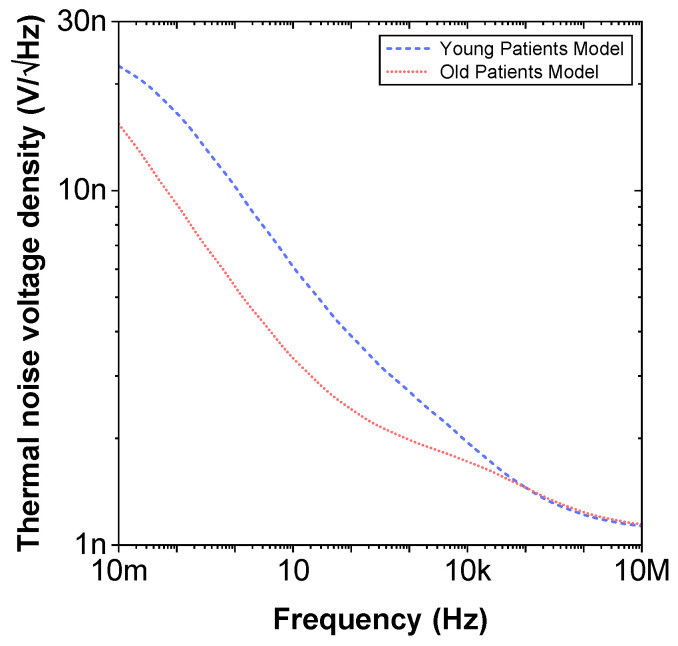
Simulated thermal noise voltage density versus frequency of proposed recurrent circuit models with EIA standard compliant RC values.

To compare the ideal recurrent model and the model with approximated CPEs, 3D Bode plots of all cases are depicted in Figure 6. The distribution of absolute relative magnitude and phase angle errors of the model with EIA standard compliant RC values is depicted in box and whisker plots with kernel density estimation curves next to 3D plots. The maximum differences between a designed magnitude/phase and a target magnitude/phase for the young and old patients model are $0.968\%/0.388^{\circ }$ and $0.918\%/0.456^{\circ }$, respectively. The distribution of absolute relative resistance and reactance errors of complex impedance of the same models (Nyquist plots depicted in Figure 3) is shown in Figure 7, in which scatterplots with contour overlay indicate the intensity of corresponding relative errors given in Table 3. The maximum differences between a designed resistance/reactance and a target [32] resistance/reactance for the young and old patients model are 1.047%/1.822% and 0.905%/1.578%, respectively. These simulations serve to highlight that the approximated CPEs using the Valsa method show good agreement with the theoretical CPE impedance and can be used in future simulation and experimental works that need to emulate the dental tissue impedance. However, they do account for variability that could be introduced based on temperature, process variations, or thermal noise. The effect of temperature on the proposed recurrent circuit models with EIA standard compliant RC values was examined in SPICE software in the range T∈{−40;+125}°C. The results of these temperature simulations are depicted in Figure 8a,b. Typical temperature coefficient values of ±200ppm/°C and ±30ppm/°C were set for resistors and capacitors, respectively, to model the linear temperature dependence of ’off the shelf’ chip components (SMDs, surface-mount devices). The calculated distribution of absolute relative magnitude and phase angle variations (top plots) show maximum ±2% change in comparison with the magnitude and phase angle responses obtained at nominal temperature $T_{\mathrm{NOM}}=27$ °C. In order to evaluate the potential parameter variation of proposed recurrent circuit models with EIA standard compliant RC values due to tolerances incurred from components manufacturing processes, Monte Carlo statistical analysis was performed for resistors and capacitors with ±1% tolerance for Gaussian distribution and 200 iterations. Figure 9a,b show the simulated magnitude and phase responses at $T_{\mathrm{NOM}}$ (bottom plots) with corresponding statistical evaluation for magnitude and phase angle at 1 Hz (histograms). Finally, the thermal noise voltage of the proposed recurrent circuit models generated by passive components was simulated. It is known, ideal capacitors, as lossless devices, do not generate thermal noise. Hence, the main source of noise in the proposed circuits is the thermal noise of the resistors, which can be expressed as $V_{R}=\sqrt{4kTBR}$ with unit $\mathrm{nV}/\sqrt{\mathrm{Hz}}$, where *k* is Boltzmann’s constant ($1.38\times 10^{-23}$ J/K), *T* is the absolute temperature in Kelvins, *B* is the bandwidth in Hertz, and *R* is the resistance value. The simulated thermal noise voltage density at $T_{\mathrm{NOM}}$ is depicted in Figure 10. The variability introduced by temperature, thermal noise, and process variation do not significantly degrade the impedance characteristics of the proposed models, supporting that they can be implemented in a wide range of conditions to support future studies needing circuits to emulate the dental tissue impedance.

## 4. Conclusions

In this work, two topologies of fractional-order equivalent electrical networks (C-C and recurrent models) have been shown to be able to represent the theoretical impedance representative of dental tissues. While both models show near-perfect agreement with each other, it highlights a limitation of using equivalent circuit modeling (not just limited to fractional-order models) that requires further investigation. That is, which model has parameters that can serve as clinically relevant biomarkers of the measured tissue. While future studies are needed to investigate the clinical utility, this work has presented additional equivalent circuit models to support researchers in the simulation and emulation of dental tissue electrical impedance using EIA standard compliant RC values. The use of the constructed model is expected to support further studies where measurements or simulations representative of dental tissues may be needed to characterize measurement equipment, but fractional-order components are not available for their realization or simulation.

## Figures and Tables

**Table 1:** Comparison of available electrical circuits employing CPEs for modeling the impedance characteristics of human teeth parts.

Tissue	Ref.	Year	Frequency Range (Hz)	Preparation of Samples	Model	
					# of Elements	Brief Description
Root Canal	[28]	2008	10–30 k	One tooth without specified eruption status and patient age.	6	Complex model employing three CPEs andthree resistors.
	[29]	2011	100–1 M	Single incisor tooth without specified eruption status and patient age.	3	Single CPE in parallel with series connection of CPE and resistor.
Enamel	[30]	1990	1–65 k	Extracted one tooth from five patients of different age groups (7 to 50 years old) with different eruption status.	5	Complex model employing single CPE, two capacitors, and two resistors.
Dentin	[31]	1992	1–65 k	Two un-erupted third molars (18 and 38) from one patient.	4	Single CPE in parallel with three resistors in series.
	[32]	2007	10 m–10 M	Five un-erupted third molars from 20 (±1) and 50 (±1) years old patients.	5	Double dispersion Cole impedance model.

**Table 2:** Parameter values for the components of the equivalent circuit models for each age groups.

Components	Elements	Cole-Cole Model Mean Values [32]		Recurrent Model Values		Recurrent Model with EIA Standard Compliant RC Values	
		*Patients*					
		*Young*	*Old*	*Young*	*Old*	*Young*	*Old*
Saline solution	*R*-ss (Ω)	71.5	72.1	71.5	72.1	71.5	72.3
Smear layer	CPE_T_-s ($\mu F\cdot \sec^{-0.5}$)	23.8	14.6	15.64	13.52	15.6	13.5
	CPE_P_-s (−)	0.5					
	*R*-s (Ω)	244	128.1	564.3	149.38	562	150
Dentins	CPE_T_-d ($\mu F\cdot \sec^{-0.5}$)	45.6	182.8	30.23	169.34	30.1	169
	CPE_P_-d (−)	0.5					
	*R*-d (kΩ)	43.1	60.9	42.78	60.88	43	60.4

**Table 3:** Comparison of results of proposed bioimpedance models fitted to data of [32].

Evaluation Criteria	Recurrent Model Values		Recurrent Model with EIA Standard Compliant RC Values	
	*Patients*			
	*Young*	*Old*	*Young*	*Old*
	**∣Re(Z) Relative Error∣ (%)**			
Max	0	0	1.047	0.905
Mean			0.330	0.345
Median			0.275	0.310
SD			0.267	0.208
	**∣−Im(Z) Relative Error∣ (%)**			
Max	0	0	1.822	1.578
Mean			0.622	0.612
Median			0.579	0.588
SD			0.395	0.400

**Table 4:** Statistical indicators of fit accuracy of proposed bioimpedance models fitted to data of [32].

Evaluation Criteria	Recurrent Model Values		Recurrent Model with EIA Standard Compliant RC Values	
	*Patients*			
	*Young*	*Old*	*Young*	*Old*
${\mathrm{MAE}}_{R}$ (Ω)	0	0	19.261	6.905
${\mathrm{MAE}}_{X}$ (Ω)			15.734	8.730
${\mathrm{RMSE}}_{R}$ (Ω)	0	0	41.744	16.591
${\mathrm{RMSE}}_{X}$ (Ω)			31.781	23.817
$R_{R}^{2}$ (−)	1	1	0.99999	0.99998
$R_{X}^{2}$ (−)			0.99991	0.99996
${\mathrm{NSE}}_{R}$ (−)	1	1	0.99997	0.99997
${\mathrm{NSE}}_{X}$ (−)			0.99989	0.99988
${\mathrm{WIA}}_{R}$ (−)	1	1	0.99999	0.99999
${\mathrm{WIA}}_{X}$ (−)			0.99997	0.99997
${\mathrm{LCE}}_{R}$ (−)	1	1	0.99659	0.99650
${\mathrm{LCE}}_{X}$ (−)			0.99394	0.99433

**Table 5:** Standard Electronic Industries Alliance (EIA) compliant RC values used in proposed CPEs of order 0.5 via the Valsa method and error analysis.

Elements	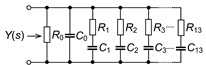 RC Network with Valsa Determined Parameters			
	*Young Patients*		*Old Patients*	
	CPE_T_-s	CPE_T_-d	CPE_T_-s	CPE_T_-d
	$15.6\;\mu F\cdot \sec^{-0.5}$	$30.1\;\mu F\cdot \sec^{-0.5}$	$13.5\;\mu F\cdot \sec^{-0.5}$	$169\;\mu F\cdot \sec^{-0.5}$
*C*_0_ (F)*/R*_0_ (Ω)	680 p/787 k	10.2 p/383 k	562 p/887 k	6.8 n/73.2 k
*C*_1_ (F)*/R*_1_ (Ω)	2.61 n/38.3	1.8 μ/6.34 k	12.1 n/232	66.5 n/7.68
*C*_2_ (F)*/R*_2_ (Ω)	6.2 n/86.6	3.74 n/12	29.4 n/536	11.8 μ/1.37 k
*C*_3_ (F)*/R*_3_ (Ω)	30 n/470	2.43 n/1.8	66.5 n/1.27 k	4.99 μ/590
*C*_4_ (F)*/R*_4_ (Ω)	13.7 n/205	8.66 n/30.9	158 n/2.94 k	28 n/3.3
*C*_5_ (F)*/R*_5_ (Ω)	909 n/13.3 k	21.5 n/73.2	365 n/6.98 k	13.7 n/1.1
*C*_6_ (F)*/R*_6_ (Ω)	42.2 μ/374 k	4.32 μ/15.4 k	35.7 μ/442 k	66.5 μ/7.68 k
*C*_7_ (F)*/R*_7_ (Ω)	1.33 n/12.7	52.3 n/180	5.23 n/100	374 n/43.2
*C*_8_ (F)*/R*_8_ (Ω)	178 n/2.4 k	309 n/1.07 k	887 n/16 k	909 n/102
*C*_9_ (F)*/R*_9_ (Ω)	14 μ/178 k	76.8 μ/187 k	2.32 n/41.2	28 μ/3.24 k
*C*_10_ (F)*/R*_10_ (Ω)	2.26 μ/29.4 k	25.5 μ/88.7 k	2.05 μ/38.3 k	158 n/18.2
*C*_11_ (F)*/R*_11_ (Ω)	75 n/1.02 k	127 n/442	1.1 n/14	2.15 μ/249
*C*_12_ (F)*/R*_12_ (Ω)	402 n/5.9 k	10.5 μ/36.5 k	12 μ/215 k	464 μ/36.5 k
*C*_13_ (F)*/R*_13_ (Ω)	5.76 μ/69.8 k	750 n/2.61 k	4.99 μ/88.7 k	154 μ/18.2 k
	**CPE_P_ Values (−)/Phase Angle (Deg.)**			
Mean	0.500/−45.001	0.500/−44.973	0.500/−44.997	0.499/−44.954
SD	0.003/0.290	0.003/0.315	0.003/0.284	0.003/0.268
∣CoV∣ (%)	0.644	0.700	0.631	0.597
	**CPE_T_ Values ($\mu F\cdot \sec^{{\mathrm{CPE}}_{P-\mathrm{mean}}}$)**			
Mean	15.615	30.087	13.430	169.856
SD	0.456	1.054	0.396	4.982
∣CoV∣ (%)	2.921	3.503	2.946	2.933
	**∣Relative Magnitude Error∣ (%)**			
Max	1.150	1.188	1.075	1.188
Mean	0.467	0.493	0.420	0.481
Median	0.424	0.474	0.384	0.459
SD	0.305	0.291	0.265	0.311
	**∣Phase Angle Error∣ (Deg.)**			
Max	0.623	0.556	0.606	0.526
Mean	0.247	0.282	0.244	0.240
Median	0.235	0.298	0.238	0.244
SD	0.153	0.143	0.145	0.129

## Abbreviations

The following abbreviations are used in this manuscript:

C-C	Double dispersion Cole impedance model
CNLS	Complex nonlinear least-squares
CoV	Coefficient of variation
${\mathrm{CPE}}_{P}$	Order of constant phase element
${\mathrm{CPE}}_{T}$	Pseudo-capacitance of constant phase element
CPE	Constant phase element
EIA	Electronic Industries Alliance
EIS	Electrical impedance spectroscopy
FOC	Fractional-order capacitor
FOE	Fractional-order element
FOI	Fractional-order inductor
LCE	Legates’s coefficient of efficiency
MAE	Mean absolute error
NSE	Nash–Sutcliffe’s efficiency
$R^{2}$	Coefficient of determination
R, X	Real, imaginary parts of the complex impedance
Rec-2	Recurrent electrical impedance model for n=2 bifurcations
RMSE	Root mean squared error
SD	Standard deviation
WIA	Willmott’s index of agreement
$\tilde{x}$	Mean value
